# Percepción de los pacientes en cuanto al manejo de la fibrosis pulmonar idiopática. Proyecto Explora-IPF

**DOI:** 10.1016/j.opresp.2022.100158

**Published:** 2022-01-17

**Authors:** Teresa Peña Miguel, Virginia Ortoll Polo, Luis Lizán, Silvia Armengol, Alba Ramón

**Affiliations:** a Servicio de Neumología, Hospital Universitario de Burgos, Burgos, España; b Servicio de Farmacia, Hospital Universitario de Burgos, Burgos, España; c Outcomes’10, SLU, Castellón, España; d Departamento de medicina, Universitat Jaume I, Castellón, España; e Boehringer Ingelheim España S.A., Sant Cugat del Vallès, Barcelona, España

**Keywords:** Fibrosis pulmonar idiopática, Calidad de vida relacionada con la salud, Preferencias, Perspectiva del paciente, Experiencia del paciente, Encuesta, Idiopathic pulmonary fibrosis, Health-related quality of life, Preferences, Patient perspective, Patient experience, Survey

## Abstract

**Introducción:**

La fibrosis pulmonar idiopática (FPI) impacta en la vida del paciente y de sus familiares, por ello, resulta necesario conocer su percepción en el abordaje de su enfermedad.

**Métodos:**

Estudio observacional mediante un cuestionario específico que recoge: variables sociodemográficas y clínicas, percepción sobre el impacto de la enfermedad (escala Likert 5 puntos), preferencias acerca de las características de la medicación (grado importancia/preocupación 1-10) y satisfacción con el tratamiento (*Treatment Satisfaction with Medicines Questionnaire* [SATMED-Q®] escala 0-100).

**Resultados:**

Participaron 69 individuos (edad: 66,5 ± 7,6 años; tiempo hasta diagnóstico: 16,5 ± 17,4 meses; tiempo de diagnóstico: 49,6 ± 42,3 meses). La mayoría indicó que la FPI les limita física (90%) y emocionalmente (75%). Las características más valoradas del tratamiento fueron: condición de enlentecer la progresión (7,4 ± 2,8), estabilizar la capacidad pulmonar (6,9 ± 2,8) y mejorar la calidad de vida (6,9 ± 2,8), por encima de mejorar/estabilizar los síntomas (6,1 ± 2,8/6,3 ± 2,8) o evitar la hospitalización (6,6 ± 2,7). El riesgo de sufrir molestias gástricas (7,1 ± 2,9), fotosensibilidad (6,6 ± 3,0) e interacciones con otros fármacos (6,0 ± 3,0) fueron los principales factores de preocupación. La satisfacción global con el tratamiento actual fue de 61,1 puntos, otorgando las puntuaciones más altas al seguimiento médico (79,5) y la opinión general sobre la medicación (74,3).

**Conclusiones:**

Estudio realizado en España que describe la perspectiva del paciente con FPI con respecto a la enfermedad y su tratamiento. Los resultados muestran un elevado nivel de concienciación acerca de la gravedad de la enfermedad por parte de los pacientes, cuyo principal interés es enlentecer su progresión. La información proporcionada puede contribuir a optimizar el manejo del paciente con FPI.

## Graphical abstract

**Figure:**
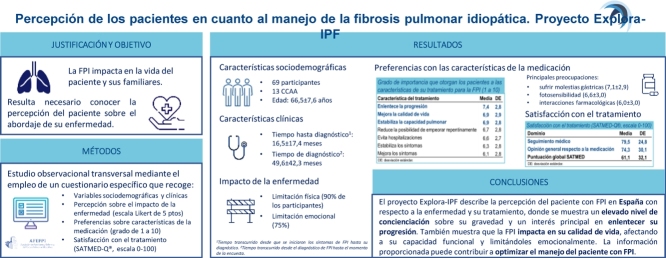

## Abstract (en)

**Introduction:**

Idiopathic pulmonary fibrosis (IPF) impacts the life of patients and their families, so it is necessary to ascertain their perception in the approach to their disease.

**Methods:**

Observational study by means of a specific questionnaire that includes socio-demographic and clinical variables, perception of the impact of the disease (5-point Likert scale), preferences regarding the characteristics of the medication (degree of importance/concern 1-10) and satisfaction with treatment (SATMED-Q® scale 0-100).

**Results:**

69 individuals participated (age: 66.5 ± 7.6 years; time until diagnosis: 16.5 ± 17.4 months; diagnosis time: 49.6 ± 42.3 months). The majority state that IPF limits them physically (90%) and emotionally (75%). The most highly valued features of the treatment were: slowing down progression of the disease (7.4 ± 2.8), stabilising lung capacity (6.9 ± 2.8) and improving quality of life (6.9 ± 2.8), above stabilising/improving symptoms (6.1 ± 2.8/6.3 ± 2.8) or avoiding hospitalisation (6.6 ± 2.7). The principal factors of concern were suffering gastric disorders (7.1 ± 2.9), photosensitivity (6.6 ± 3.0) or interaction with other drugs (6.0 ± 3.0). Overall satisfaction with the current treatment scored 61.1 points, with the highest scores being for medical follow-up (79.5) and overall opinion of the medicine (74.3).

**Conclusions:**

A study conducted in Spain on the perspective of IPF patients regarding the disease and its treatment. The results show a high level of awareness in terms of the seriousness of the disease on the part of patients, whose main concern is to slow down its progression. The information provided may help to optimise the management of IPF patients.

## Introducción

La fibrosis pulmonar idiopática (FPI) es la forma más común de las enfermedades pulmonares intersticiales difusas (EPID)^1^. Se trata de una enfermedad rara con una incidencia estimada de 7,4 afectados por cada 100.000 habitantes en mujeres y de 10,7 en hombres, y una prevalencia estimada de 13/100.000 y 20/100.000 en mujeres y hombres, respectivamente^2^. De acuerdo con ello, en España se estima que podría haber unos 7.500 afectados^3^. Es una enfermedad crónica, mortal y altamente incapacitante, caracterizada por un deterioro progresivo de la función pulmonar debido a un proceso de cicatrización anormal e irreversible del tejido pulmonar^2^, ^4^. La etiología se desconoce, aunque probablemente se deba al efecto de diversos factores (tabaquismo, autoinmunidad, infecciones víricas y ciertos factores medioambientales) en sujetos con predisposición genética^5^, ^6^.

La FPI se asocia con un mal pronóstico, ya que presenta una evolución hacia la insuficiencia respiratoria, con una esperanza media de vida de solo tres a cuatro años en los pacientes que no reciben tratamiento antifibrótico^7^. Aunque no existe un tratamiento curativo, en los últimos años, se ha avanzado enormemente en el desarrollo de teapias para frenar su progresión^8^. En este sentido, en el año 2017 se publicó la «Normativa sobre el tratamiento farmacológico de la fibrosis pulmonar idiopática»^9^, que recoge la actualización del consenso internacional sobre FPI^10^, donde se establece la recomendación terapéutica de dos fármacos antifibróticos, nintedanib y pirfenidona, por haber demostrado una disminución significativa de la función pulmonar^9^, ^11^. Además de las opciones farmacológicas, en algunos casos se recomienda la oxigenoterapia domiciliaria a largo plazo y/o la rehabilitación pulmonar^12^.

Debido al considerable impacto de la FPI en la calidad de vida relacionada con la salud (CVRS), resulta necesario abordar la enfermedad teniendo en cuenta las experiencias y expectativas del paciente, con el fin de mejorar el manejo clínico y su satisfacción^13^. En Europa, se han llevado a cabo diversas iniciativas basadas en entrevistas con pacientes para dar a conocer sus necesidades durante el proceso asistencial y el impacto de la FPI en su CVRS^14^, ^15^, ^16^. Sin embargo, los datos que existen a nivel nacional son escasos y prácticamente se limitan a los resultados de un estudio cualitativo realizado con 45 pacientes de cinco países europeos (incluyendo nueve pacientes españoles), analizados de forma conjunta y publicados en 2011^17^. Es necesario, por tanto, ampliar y actualizar los datos clínicos y sociodemográficos de los pacientes con FPI en España, y poner en valor su perspectiva respecto a la enfermedad y su tratamiento. Así pues, el objetivo de este trabajo es dar a conocer la percepción del paciente con FPI en relación con el manejo terapéutico de su enfermedad dentro del ámbito sanitario español. Específicamente, se pretende describir la percepción del paciente acerca del impacto de la FPI en su vida diaria, conocer sus preferencias sobre las características de los distintos tratamientos farmacológicos, así como su satisfacción con el tratamiento y la información recibida.

## Métodos

Estudio observacional transversal basado en un cuestionario *on-line* dirigido a pacientes adultos, con diagnóstico de FPI, o bien, a sus familiares o cuidadores. En caso de tratarse de un familiar o cuidador, el cuestionario debía responderse desde la perspectiva del paciente. La Asociación de Familiares y Enfermos de Fibrosis Pulmonar Idiopática (AFEFPI) fue la responsable de invitar a participar a sus asociados mediante correo electrónico. Fue requisito indispensable que el participante otorgara su consentimiento para participar en el estudio.

El cuestionario se desarrolló específicamente (*ad hoc*) para el estudio a partir de la revisión de la literatura sobre los aspectos de interés relacionados con el abordaje de la FPI, contando con la participación de una neumóloga y una farmacéutica hospitalaria como expertas en el manejo de la FPI. Miembros de la AFEFPI revisaron el cuestionario con el fin de asegurar su comprensibilidad. El estudio cuenta con la aprobación del Comité Ético de Investigación con Medicamentos del Área de salud de Burgos y Soria.

El cuestionario incluía 18 ítems distribuidos en cinco apartados (Appendix A, Tabla A.1): 1) variables sociodemográficas (edad, sexo, provincia de residencia, nivel de estudios, situación laboral, situación familiar/convivencia), 2) variables clínicas y relacionadas con el tratamiento y el seguimiento de la FPI (tiempo desde diagnóstico, tiempo hasta diagnóstico, tipo de tratamiento, profesionales sanitarios implicados en el manejo de la enfermedad), 3) información recibida acerca de la enfermedad y tratamiento general (tipo de información recibida, profesional sanitario que informa sobre la FPI, profesional sanitario que informa sobre el tratamiento), 4) percepción de la enfermedad (escala Likert de cinco puntos: totalmente en desacuerdo a totalmente de acuerdo que valora cómo impacta la FPI en el día a día, físicamente, emocionalmente, a la vida social, a la calidad del sueño o a las actividades cotidianas del paciente) y 5) preferencias sobre los tratamientos farmacológicos (escala de calificación numérica de 1 a 10 y ejercicio de ordenación donde el participante indica: a) la importancia otorgada a que el tratamiento mejore la calidad de vida, enlentezca la progresión de la enfermedad, estabilice la capacidad pulmonar, mejore los síntomas, estabilice los síntomas, reduzca la posibilidad de empeoramiento repentino de la enfermedad y evite hospitalizaciones; y b) su preocupación por tener que aumentar la dosis, tomar un número elevado de pastillas, la interacción con otros fármacos o alimentos, aparición de molestias gastrointestinales o fotosensibilidad). Al final del cuestionario se incluía también la versión traducida al español del cuestionario *Treatment Satisfaction with Medicines Questionnaire* (SATMED-Q®) de satisfacción con el tratamiento farmacológico actual. Este cuestionario se divide en seis dominios: eficacia del tratamiento, comodidad de uso de la medicación, impacto de la medicación en actividades cotidianas, atención médica, opinión general respecto a la medicación y efectos indeseables de la medicación^18^.

Para el análisis descriptivo de la muestra, se calcularon las frecuencias relativas y absolutas para describir las variables cualitativas, mientras que para las variables cuantitativas se calcularon los estadísticos de centralidad y dispersión (media, desviación estándar [DE], mínimo y máximo). El orden de importancia se presenta de forma decreciente según la puntuación de importancia obtenida (calculada como el sumatorio de las puntuaciones). Todos los resultados obtenidos se calcularon respecto a las respuestas válidas otorgadas a cada una de las preguntas, informándose en cada caso del número de datos (N) sobre los que se realizaron los cálculos. En el caso de las variables sociodemográficas referentes a la edad, el género, el nivel de estudios y la situación laboral, solo se consideraron las respuestas de los pacientes, ya que algunos familiares/cuidadores respondieron la pregunta de forma personal.

### Consideraciones éticas

El estudio fue aprobado por el Comité de Ética de IESCYL de Castilla y león. Los autores confirman que todos los participantes otorgaron su consentimiento para participar en el estudio.

## Resultados

### Características sociodemográficas

La encuesta fue respondida por un total de 69 participantes: 46 pacientes (66%) y 23 familiares/cuidadores (33%). La media de edad de los pacientes fue de 66,5 años (DE: 7,6, rango: 49-80, n = 46), de los cuales el 85% fueron hombres (n = 39).

Se obtuvo representación de pacientes de 13 Comunidades Autónomas, siendo la Comunidad de Madrid (29%) la que contaba con la mayor representación (tabla 1). El 97% de los pacientes (n = 67) vivía acompañado por otra persona, únicamente dos sujetos vivían solos. La mayoría de los pacientes (76%) tenía estudios secundarios o universitarios y casi un 70% estaba jubilado.

**Tabla 1:** Nivel de estudios, situación laboral y lugar de residencia

Característica sociodemográfica	n (%)
Nivel de estudios (n = 46)	
Sin estudios	1 (2,2)
Primaria	8 (17,4)
Secundaria	13 (28,3)
Estudios universitarios	20 (43,5)
Estudios de tercer ciclo	2 (4,3)
Otros^a^	2 (4,3)
Situación laboral (n = 46)	
Trabajador en activo	7 (15,2)
Jubilado	32 (69,6)
Incapacidad laboral	3 (6,5)
Incapacidad laboral debido a la FPI	3 (6,5)
Otros^b^	1 (2,2)
Lugar de residencia (n = 69)	
Andalucía	12 (17,4)
Aragón	8 (11,6)
Asturias	2 (2,9)
Castilla La Mancha	2 (2,9)
Castilla y León	3 (4,3)
Cantabria	1 (1,4)
Cataluña	7 (10,1)
Comunidad Valenciana	6 (8,7)
Extremadura	3 (4,3)
Islas Baleares	2 (2,9)
Islas Canarias	1 (1,4)
Madrid	20 (29,0)
País Vasco	2 (2,9)

a Estudios de comercio (n = 1), carrera militar (n = 1).

b Baja laboral, sin especificar motivo.

### Características clínicas y relacionadas con el tratamiento

La media de tiempo de diagnóstico fue de 49,6 meses (DE: 42,3, rango: 3-229, n = 69), y el tiempo medio transcurrido desde la aparición de los síntomas hasta que se les diagnosticó la enfermedad fue de 16,5 meses (DE: 17,4; mediana: 12, rango: 0-96, n = 67). La mayoría de los pacientes habían sido diagnosticados en un tiempo inferior o igual al año. Además del tratamiento farmacológico, un 43,5% (n = 30) de los pacientes recibía tratamiento no farmacológico (oxigenoterapia domiciliaria, rehabilitación respiratoria u otros). El seguimiento de todos los pacientes estaba a cargo del neumólogo y, en un 40% de los casos, estos no visitaban ningún otro profesional sanitario en relación con la FPI (tabla 2).

**Tabla 2:** Características clínicas y personal sanitario involucrado en el seguimiento de los pacientes con fibrosis pulmonar idiopática

Característica	n (%)
*Tiempo transcurrido desde el diagnóstico*	
≤ 3 años	32 (46,4)
3-5 años	25 (36,2)
> 5 años	12 (17,4)

*Tiempo desde la aparición de los síntomas hasta el diagnóstico*	
≤ 1 año	41 (61,2)
1-2 años	14 (20,9)
2-3 años	5 (7,5)
≥ 3 años	7 (10,4)

*Profesionales sanitarios*	
Neumólogo	69 (100)
Médico de atención primaria	28 (40,6)
Farmacia hospitalaria	19 (27,5)
Enfermería	12 (17,4)
Radiólogo	11 (15,9)
Cuidados crónicos/paliativos	4 (5,8)
Anatomía Patológica	1 (1,4)
Otros^a^	7 (10,1)

a Reumatólogo (n = 1), unidad de trasplante (n = 2), unidad de cirugía torácica (n = 1), psicólogo (n = 1), nutricionista (n = 1), sin especificar (n = 1).

### Información recibida acerca de la enfermedad y los tratamientos

**Figura 1:**
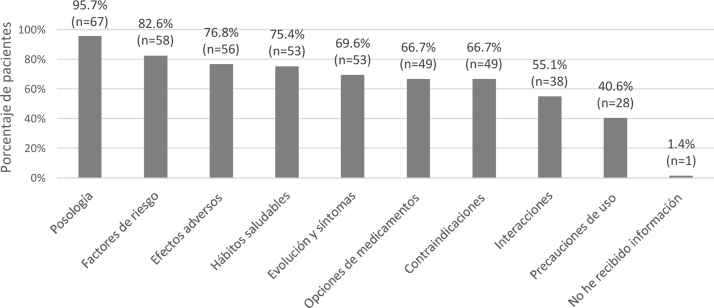
Información recibida sobre la fibrosis pulmonar idiopática y su tratamiento según las respuestas de los pacientes (n = 69).

La mayoría de los pacientes (> 75%) habían recibido información sobre dosis y frecuencia de administración del tratamiento, factores de riesgo de la FPI, hábitos saludables y efectos adversos. En más de la mitad de los casos (55-70%), también habían sido informados acerca de la evolución y síntomas de la enfermedad, opciones de tratamiento, contraindicaciones e interacciones con otros fármacos. Menos de la mitad de los pacientes había recibido información sobre las precauciones de uso del fármaco. Solamente un sujeto, indicó no haber recibido ninguna información (fig. 1).

En la mayoría de los casos, el neumólogo fue el profesional sanitario que proporcionó información a los pacientes acerca de la enfermedad y su tratamiento (84 y 96%, respectivamente). En el resto de los casos, la información acerca de la enfermedad la consiguieron principalmente a través de la asociación de pacientes (n = 4), de Internet (n = 3), atención primaria (n = 1), enfermería (n = 1), psicólogo (n = 1) o por sus medios (n = 1); y, la información sobre el tratamiento, del médico de atención primaria (n = 1), del personal de enfermería (n = 1) o del personal de la unidad de cuidados paliativos (n = 1).

### Percepción sobre la enfermedad

**Figura 2:**
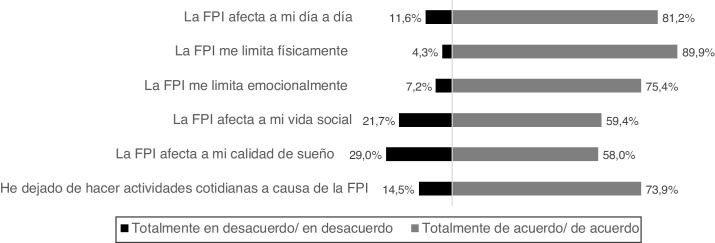
Grado de acuerdo con las afirmaciones de percepción sobre la enfermedad (n = 69). FPI: fibrosis pulmonar idiopática. Porcentaje agrupado para el acuerdo y el desacuerdo, el porcentaje restante corresponde a la respuesta «ni de acuerdo ni en desacuerdo» (no representado).

La mayoría de los participantes señaló que la FPI les limita físicamente (58%, n = 40 pacientes indicaron estar totalmente de acuerdo y 32%, n = 22 de acuerdo), afecta a su día a día (65%, n = 45 totalmente de acuerdo y 16%, n = 11 de acuerdo), les impide realizar las actividades cotidianas (46%, n = 32 totalmente de acuerdo y 28%, n = 19 de acuerdo) y les limita emocionalmente (36%, n = 25 totalmente de acuerdo y 39%, n = 27 de acuerdo). El 60% de los pacientes también indicó que la FPI afectaba a su vida social (26%, n = 18 totalmente de acuerdo y 33%, n = 23 de acuerdo). El 58% de los pacientes (25%, n = 17 totalmente de acuerdo y 33%, n = 23 de acuerdo) indicaron que la enfermedad afectaba a su calidad del sueño (fig. 2).

### Percepción sobre los tratamientos farmacológicos

En cuanto a la percepción sobre los tratamientos farmacológicos, si bien todas las otras características propuestas fueron valoradas como importantes, la característica con mayor grado de importancia para los pacientes fue que el tratamiento enlentezca la progresión de la enfermedad (tabla 3).

**Tabla 3:** Grado de importancia y clasificación por orden decreciente de importancia que otorgan los pacientes con fibrosis pulmonar idiopática (n = 69) a las características de su tratamiento

Característica del tratamiento	Grado de importancia^a^ (media ± DE)	Clasificación por orden de importancia^b^
Enlentece la progresión de la enfermedad	7,4 ± 2,8	1
Estabiliza la capacidad pulmonar	6,9 ± 2,8	2
Mejora la calidad de vida	6,9 ± 2,9	3
Reduce la posibilidad de sufrir un empeoramiento repentino	6,7 ± 2,8	4
Estabiliza los síntomas	6,3 ± 2,8	5
Mejora los síntomas	6,1 ± 2,8	6
Evita hospitalizaciones	6,6 ± 2,7	7

a Escala del 1 al 10.

b Cálculo basado en el sumatorio de todas las respuestas obtenidas en la pregunta de ordenación por importancia. DE: desviación estándar.

### Miedos y preocupaciones relacionados con el tratamiento farmacológico para la FPI

La característica del tratamiento que preocupaba más a los pacientes fue que pudiera producir molestias gástricas leves (náuseas, diarrea), seguida de la posibilidad de sufrir una reacción de fotosensibilidad (sensibilidad solar que puede producir algunas reacciones en la piel) y la interacción con otros fármacos y/o alimentos. Otras características del tratamiento como el número elevado de pastillas y tener que aumentar la dosis del tratamiento con el tiempo también preocupaban a los pacientes, pero en menor medida (tabla 4).

**Tabla 4:** Grado de preocupación o miedo y clasificación por orden decreciente de importancia que otorgan los pacientes de fibrosis pulmonar idiopática (n = 69) a las características de su tratamiento

Característica del tratamiento	Grado de preocupación/miedo^a^ (media ± DE)	Clasificación por orden de importancia^b^
Que el tratamiento me produzca molestias gástricas leves	7,1 ± 2,9	1
Que el tratamiento interaccione con otros fármacos	6,0 ± 3,0	2
Que el tratamiento me produzca reacciones de fotosensibilidad	6,6 ± 3,0	3
Que el tratamiento interaccione con algunos alimentos	5,6 ± 3,0	4
Tener que aumentar la dosis del tratamiento con el tiempo	5,3 ± 3,1	5
Tener que tomar al día un número elevado de pastillas (> 5)	5,0 ± 3,1	6

a Escala del 1 al 10.

b Cálculo basado en el sumatorio de todas las respuestas obtenidas en la pregunta de ordenación por preocupación. DE: desviación estándar.

### Satisfacción con el tratamiento farmacológico actual

La puntuación global del cuestionario de satisfacción con la medicación SATMED-Q® fue de 61,1 sobre 100 (tabla 5), una mayor puntuación indicaba un mayor grado de acuerdo o satisfacción con las afirmaciones propuestas. El dominio mejor valorado fue el relacionado con el seguimiento médico, seguido de la opinión general con la medicación y la comodidad de esta.

**Tabla 5:** Resultados del cuestionario SATMED-Q por dominios y global

Dominio	Media	DE	n
Efectos indeseables de la medicación (0-100)^a^	55,4	32,9	57
Eficacia de la medicación (0-100)	47,2	30,5	69
Comodidad de la medicación (0-100)	71,1	26,4	69
Impacto de la medicación en la vida cotidiana (0-100)	44,4	30,6	69
Seguimiento médico (0-100)	79,5	24,8	69
Opinión general respecto a la medicación (0-100)	74,3	30,1	69
*Puntuación global SATMED (0-100)*	*61,1*	*32,1*	*-*

a Para conocer si los efectos adversos interferían en la vida de los pacientes, en este dominio solo se tuvieron en cuenta las respuestas de los pacientes que habían sufrido efectos secundarios (n = 57; 82,6%). DE: desviación estándar; SATMED-Q: *Treatment Satisfaction with Medicines Questionnaire*.

## Discusión

Los resultados del presente trabajo proporcionan información muy relevante para conocer y comprender mejor la perspectiva del paciente con FPI acerca de su enfermedad. Además, aporta datos novedosos sobre las preferencias del paciente acerca del tratamiento y su grado de satisfacción con el manejo terapéutico dentro del ámbito sanitario español.

Una de las fortalezas de este estudio es la elevada participación de pacientes. La media de edad de los participantes concuerda con lo esperado, tratándose de personas con FPI, diagnosticada habitualmente en individuos mayores de 50 años^2^. Sin embargo, la proporción de hombres obtenida (5,5:1 para el varón) es superior a la referida en la literatura, situándose esta entre 1,6:1 y 2:1^19^, ^20^. En relación con ello, Caro et al.,^21^ sugieren que la proporción de mujeres en otros estudios, previos a la publicación de los criterios diagnósticos actuales, podría estar sobreestimada, ya que las enfermedades autoinmunes son más frecuentes en el sexo femenino^22^ y pueden presentar un patrón de neumonía intersticial usual indistinguible de la FPI^23^. Para actualizar esta ratio, los mismos autores realizaron un estudio retrospectivo de una cohorte, utilizando las recomendaciones de diagnóstico actuales y obtuvieron una representación de un 24,4% de mujeres en una muestra de 86 pacientes con FPI^21^. Estos datos están más alineados con los obtenidos en el presente estudio.

El diagnóstico de la FPI es complejo y a menudo se tardan meses o incluso años en diagnosticarla correctamente^24^. Este retraso provoca ansiedad y frustración en los pacientes, conscientes del tiempo transcurrido desde que aparecen los primeros síntomas hasta que se les diagnostica la enfermedad^14^, ^15^, ^17^. Según los encuestados en este estudio, más de la mitad de los pacientes fueron diagnosticados antes de que transcurriera un año desde el inicio de los síntomas. Sin embargo, un número todavía elevado de pacientes (39%) había vivido más de un año con la enfermedad sin diagnosticar. Este retraso en el diagnóstico supone un inicio tardío del tratamiento que enlentece la progresión de la patología y, por consiguiente, los pacientes pueden presentar un deterioro de la enfermedad. Es necesario, por tanto, favorecer el diagnóstico temprano, que requiere de la exclusión de otras EPID, así como de la presencia de un patrón radiológico y/o histológico de neumonía intersticial usual^2^, ^3^. Además, es fundamental que los médicos de atención primaria estén suficientemente formados sobre la patología para tener una sospecha diagnóstica y que los pacientes puedan ser rápidamente derivados al neumólogo especialista en FPI^25^. En este sentido, los pacientes que informan de disnea que no responden a terapia con inhaladores y con crepitación pulmonar deben ser considerados para una radiografía de tórax. Este tipo de pruebas, sencillas y disponibles en práctica clínica habitual, deberían permitir la identificación y derivación temprana de un grupo de riesgo susceptible de padecer FPI^4^.

Otra de las demandas de los profesionales especialistas en FPI^26^, que también se deriva de las experiencias de los pacientes^14^, ^17^, es la existencia de unidades de expertos organizados en equipos multidisciplinares en los que participen neumólogos, radiólogos, patólogos, psicólogos y personal de enfermería para atender de manera integral las necesidades de los afectados por FPI. Además, un abordaje multidisciplinar permitiría clarificar y consensuar hallazgos clínicos, radiológicos y anatomopatológicos para actuar en estadios cada vez más tempranos de la enfermedad^9^. No obstante, a la vista de los resultados, este parece ser un objetivo que todavía no se ha alcanzado, ya que una proporción considerable de pacientes afirmó ser atendida únicamente por el neumólogo y, además del médico de atención primaria y del farmacéutico hospitalario, en muy pocos casos apareció la figura de enfermería, o psicología, entre otros, durante su seguimiento.

A pesar de ello, los resultados obtenidos en este estudio muestran importantes avances con respecto a otras encuestas previas realizadas con pacientes. Según los resultados de un estudio publicado en 2016, la mayoría de los pacientes se sentía insatisfecha con la información recibida y aproximadamente un tercio apenas conocía la enfermedad^15^. La falta de información y de educación sobre la FPI también se ha observado en otros estudios, dentro^16^, ^17^ y fuera de Europa^27^. Sin embargo, en este trabajo la gran mayoría de los encuestados afirma haber recibido información tanto acerca de la enfermedad como sobre su tratamiento y valoran la información recibida por parte de su médico (neumólogo en la mayoría de los casos) de manera muy satisfactoria. Además, este hecho pone de manifiesto el elevado conocimiento de los pacientes encuestados acerca de la FPI que, de esta forma, pueden entender y sobrellevar mejor la enfermedad.

Este estudio también recoge información relevante sobre la percepción del paciente acerca del impacto de la FPI en su vida diaria. La repercusión de esta enfermedad en la CVRS de las personas que la padecen es evidente y las funciones más afectadas son aquellas relacionadas con la actividad física. De acuerdo con ello, la mayoría de los participantes señaló que la FPI les limitaba físicamente, afectando a su día a día e impidiéndoles realizar las actividades cotidianas. Además, esta incapacidad para realizar las rutinas se relaciona con un efecto negativo sobre su bienestar emocional^16^, ^17^. El impacto en la vida social y en la calidad del sueño, aunque mayoritario entre los participantes, podría estar asociado con los estadios más avanzados de la enfermedad^28^, ^29^. La mala calidad del sueño se relaciona con la gravedad de los trastornos respiratorios durante el descanso nocturno que pueden variar en función de las características del paciente^30^. El curso clínico de la enfermedad es muy variable, tanto entre pacientes como entre periodos en un mismo individuo, y su evolución depende de diversos factores como la edad, las complicaciones o las comorbilidades asociadas^26^. Se trata, por tanto, de una enfermedad con alto nivel de impacto en la CVRS, que va empeorando conforme la patología avanza. Por ello, mantener a los pacientes el máximo tiempo posible en estadios menos avanzados (función pulmonar más preservada) resulta fundamental.

Este es el primer estudio que investiga la percepción del paciente acerca de los tratamientos farmacológicos actuales en España. De ellos, se infiere que el principal interés de los pacientes, conscientes de la gravedad de la FPI y conocedores de las opciones de tratamiento, sea que la medicación enlentezca la progresión de la enfermedad. La mayor preocupación de los pacientes sobre los tratamientos farmacológicos es que produzcan molestias gástricas leves, reacciones de fotosensibilidad y que interaccionen con otros fármacos. Nintedanib y pirfenidona son los dos fármacos antifibróticos aprobados para el tratamiento de pacientes con FPI^9^. Ambos difieren en su perfil de seguridad^31^, ^32^ con la fotosensibilidad como efecto adverso (EA) específico de pirfenidona, pero comparten las molestias gástricas leves como el EA más común. En el caso de nintedanib, los EA gastrointestinales más frecuentes son la diarrea, náuseas y dolor abdominal^33^, mientras que con pirfenidona los más frecuentes son náuseas, dispepsia y diarrea^34^. La gran mayoría de los participantes (82,6%) había sufrido EA producidos por la medicación y, de acuerdo con el cuestionario SATMED®, a más de la mitad les había interferido en sus tareas cotidianas, actividad física y tiempo libre. La preocupación de los pacientes sobre las interacciones farmacológicas varía entre ambos medicamentos y, por tanto, obtuvo más variabilidad de respuesta, pues dependen de la opción que conozca el paciente y sus circunstancias personales (medicación concomitante y/o preferencias alimentarias).

La opinión global respecto a la medicación recibida fue considerablemente buena, puesto que la mayoría de los pacientes tenía intención de continuar con el tratamiento, se sentía a gusto con él y, en general, estaban satisfechos. Además, consideran cómodo su modo y frecuencia de administración. A pesar de ello, la mayoría no refiere encontrarse mejor de lo que se encontraba antes de iniciar el tratamiento, ni parece que la medicación les haya ayudado a realizar mejor sus actividades diarias, de ocio y tiempo libre, o sus tareas de aseo personal. Los tratamientos empleados para la FPI ralentizan la progresión del deterioro de la función pulmonar, pero no consiguen revertirla, por ello, es difícil que el paciente pueda percibir una mejora de su sintomatología asociada al tratamiento.

El estudio presenta una serie de limitaciones inherentes a su diseño y a la población de estudio. El uso de preguntas *ad hoc* en el cuestionario puede representar una restricción. Asimismo, el cuestionario incluía únicamente preguntas con respuestas cerradas, lo que puede dificultar la interpretación de las percepciones de los participantes, no permitiéndoles, si fuera necesario, matizar su respuesta. Hay que resaltar que el cuestionario fue revisado y validado por dos profesionales sanitarias expertas en el manejo de la FPI y por pacientes, asegurando de este modo su idoneidad y comprensibilidad. Por otro lado, con relación a la población de estudio, cabe señalar que puede existir un sesgo de selección, ya que los participantes pertenecían a una asociación de pacientes, por lo que podrían tener un mayor conocimiento sobre la FPI y estar más involucrados en su manejo que los no asociados y, por tanto, su perspectiva podría variar. A pesar de las limitaciones, los datos que se derivan de este trabajo tienen una gran relevancia para comprender aquellos factores que pueden contribuir a mejorar la satisfacción y el cumplimiento con el tratamiento y, con ello, los resultados en salud.

Este es el primer estudio que se realiza en España que pone de manifiesto la perspectiva del paciente con FPI respecto al manejo terapéutico de su enfermedad. Los resultados muestran un elevado nivel de concienciación acerca de la gravedad de la enfermedad por parte de los pacientes, cuyo principal interés es enlentecer la progresión de la misma. Asimismo, dado que se trata de una enfermedad con un alto impacto en la CVRS del paciente, conocer la opinión de quienes la padecen es fundamental para mejorar su manejo. En este sentido, la información obtenida en este estudio, del que destaca su elevada participación, puede contribuir de forma considerable a optimizar el abordaje de la FPI.

## Financiación

El estudio EXPLORA-IPF ha sido financiado por Boehringer Ingelheim International GmbH (BI).

## **Conflicto de** intereses

Los autores cumplen con los criterios de autoría según lo recomendado por el International Committee of Medical Journal Editors (ICMJE). Los autores no recibieron ningún pago para el desarrollo del artículo. T. Peña Miguel ha impartido charlas para las siguientes empresas farmacéuticas en los últimos años: GlaxoSmithKline, Ferrer, Novartis, Menarini, Astra Zeneca, Chiesi, BI y Roche. V. Ortoll Polo declara no tener conflicto de interés. L. Lizán fue contratado y financiado por BI. S. Armengol y A. Ramón son empleadas de BI. Laura Benedito-Palos, trabajadora de una consultoría científica y estratégica independiente (Outcomes’10), ha recibido honorarios por su contribución al desarrollo y coordinación del proyecto y a la redacción de este manuscrito.

## Agradecimientos

Los autores agradecen a todos los miembros de AFEFPI que han participado en el estudio por su valiosa colaboración.

## Footnotes

Anexo Se puede consultar material adicional a este artículo en su versión electrónica disponible en doi:10.1016/j.opresp.2022.100158.
